# Label-free and rapid mechanics of single cells under high-density co-culture conditions by deep learning image recognition-assisted atomic force microscopy: Cell mechanics by deep learning image recognition-assisted AFM

**DOI:** 10.3724/abbs.2024158

**Published:** 2024-09-18

**Authors:** Xuliang Yang, Mi Li

**Affiliations:** 1 State Key Laboratory of Robotics Shenyang Institute of Automation Chinese Academy of Sciences Shenyang 110016 China; 2 School of Artificial Intelligence Shenyang University of Technology Shenyang 110870 China

Mechanical cues play an important role in regulating cellular activities. Cells are able to sense and respond to the mechanical cues present in the extracellular physical microenvironment via mechanotransduction [1], which can ultimately shape the functions and behaviors of the cells themselves as well as their microenvironments during numerous developmental, physiological and pathological processes [2]. The development of human diseases such as cancer is generally accompanied by unique changes in the mechanical properties of cells and their physical microenvironments, and discoveries in the field of physical oncology are beginning to be translated into new therapeutic strategies for cancer [3]. Delineating the mechanical properties of biological tissues in various dimensions from individual cells to organs is therefore fundamental [4] for dissecting the mysteries of life and advancing human healthcare. In particular, atomic force microscopy (AFM)-based force spectroscopy [5] has become a powerful, standard and multifunctional toolbox for characterizing the various mechanical properties of single cells at the micro/nanoscale. However, current studies of AFM-based single-cell mechanical measurements rely mainly on the experience of the experimental operator to move the AFM probe to the target cells for subsequent force measurements, which often results in a time-consuming and laborious experimental process. In addition, cell coculture has been widely used in the field of life sciences to examine intercellular interactions. Nevertheless, in current cell coculture studies [6], cells are commonly labelled with fluorescent molecules so that one can visually identify the specific cell types in the coculture, which can affect the behaviors of the fluorescently labelled cells. Consequently, developing a method that allows AFM to measure the mechanical properties of cells under coculture conditions in an efficient and fluorescence-independent way will significantly benefit the applications of AFM in the field of mechanobiology.

Previously, we presented a method based on the combination of AFM and deep learning optical image recognition [7], which can precisely move the AFM probe to individual targeted cells to perform mechanical measurements under low-density co-culture conditions (nearly no contact between different cell types in the co-culture). Here, we present a study of deep learning image recognition-assisted AFM to rapidly probe the mechanical properties of single living cells grown in high-density co-culture conditions (with different cell types in contact with each other in the co-culture) without the need for fluorescent labelling. In this work, AFM experiments were performed with a commercial JPK NanoWizard AFM (Bruker, Santa Barbara, USA), which was mounted on an inverted optical microscope (Nikon, Tokyo, Japan). Three types of cells, MGC-803 (a human gastric cancer cell line), HGC-27 (a human undifferentiated gastric cancer cell line), and HMrSV5 (a human peritoneal mesothelial cell line), were used. All three types of cells were cultured in RPMI-1640 medium supplemented with 10% fetal bovine serum (FBS) and 1% penicillin-streptomycin at 37°C (5% CO _2_ and 95% air) in Petri dishes. During the experiments, the RPMI-1640 medium was replaced by CO _2_-independent Leibovitz’s L-15 medium, and the AFM experiments were performed at 37°C (the commercial AFM used here has a heater system). MGC-803 cells (stained with the DiI dye) were co-cultured with HGC-27 cells (stained with the DiO dye). Both optical bright-field images and corresponding fluorescent images of co-cultured cells were recorded. The YOLOX deep learning neural network was used for directly recognizing cell types from optical bright-field images. The fluorescent images were used to assist in the preparation of the training datasets ( Supplementary Figure S1) and to verify the detection results of the deep learning image recognition model. In a previous study under low-density co-culture conditions [7], we reduced the complexity of the YOLOX neural network to improve the detection speed without reducing the detection accuracy. Under high-density co-culture conditions, where cell recognition becomes much more difficult, we found that reducing the complexity of the YOLOX model resulted in decreased detection accuracy. We examined the recognition performances of four YOLO series neural networks (YOLOX, YOLOv5, YOLOv7, and YOLOv8), and the experimental results revealed that the YOLOX model had the highest detection precision (89.25%) ( Supplementary Table S1) and the best detection result ( Supplementary Figure S2) and could meet the experimental requirements. Hence, the YOLOX neural network was used here. The AFM spherical probe (a microsphere attached to the tipless cantilever) was used in the indentation assay to measure the Young’s modulus of the cells, and the AFM single-cell probe (a living HMrSV5 cell attached to the tipless cantilever) was used in the single-cell force spectroscopy (SCFS) assay to measure the adhesion force of the cells. More experimental details ( *e*. *g*., cell sample preparation, deep learning image recognition model, AFM spherical probe preparation, single-cell probe preparation, AFM experiments, and data analysis) are provided in the Supplementary Materials.

The experimental results show that deep learning image recognition-assisted AFM allows AFM-based force spectroscopy to automatically and effectively measure the mechanical properties of single living high-density co-cultured cells on the basis of the fluorescence-independent identification of cell types. Here, high-density co-culture means that different cell types in the co-culture are in physical contact with each other and that the total number of cells covers approximately 60% or more of the bottom surface of the culture dish. In contrast to high-density co-culture, low-density co-culture refers to co-culture in which different cell types barely touch each other and total cells cover approximately 10% or less of the culture dish. Figure 1 shows the results of measuring the Young’s modulus of high-density co-cultured cells by deep learning image recognition-assisted AFM. The YOLOX deep learning model accurately identified the co-cultured MGC-803 and HGC-27 cells as well as the AFM spherical probe directly from the optical bright-field image ( Figure 1A). The corresponding fluorescent image ( Figure 1B) confirmed the correctness of the detection results obtained with the YOLOX model. On the basis of deep learning model recognition, the positional relationships between the AFM spherical probe and the target cells were automatically generated (I in Figure 1D,E). The AFM spherical probe ( Figure 1C) was subsequently moved precisely to the central area of individual co-cultured MGC-803 cells (II in Figure 1D) and HGC-27 cells (II in Figure 1E) to perform an indentation assay, during which force curves were recorded to obtain the cellular Young’s modulus (III in Figure 1D,E). For each cell, 10 force curves were recorded at the same location on the cell surface. The real-time process of the automated indentation assay by deep learning image recognition-assisted AFM is shown in Supplementary Movie S1. On the basis of the established procedure, 50 MGC-803 cells and 50 HGC-27 cells were measured, and the statistical results (IV in Figure 1D,E) revealed that the Young’s modulus of HGC-27 cells was greater than that of MGC-803 cells (statistical results for each cell are shown in Supplementary Figure S3). In addition to the indentation assay, which uses a conventional AFM probe to measure the Young’s modulus of cells, the SCFS assay, which uses a biosensitive AFM probe functionalized with a living cell, has also been used as an important AFM-based force spectroscopy method for characterizing the adhesion forces of single cells [8]. Therefore, we then utilized deep learning image recognition-assisted AFM to perform an SCFS assay on high-density co-cultured cells, and the results are shown in Figure 2. To perform the SCFS assay by deep learning image recognition-assisted AFM, living HMrSV5 cells were added to dishes containing co-cultured MGC-803 cells and HGC-27 cells, and HMrSV5 cells were attached to the AFM tipless cantilever to prepare a single-cell probe ( Supplementary Figure S4). The YOLOX model was subsequently used to directly recognize the co-cultured MGC-803 cells and HGC-27 cells as well as the AFM single-cell probe in the recorded optical bright-field image ( Figure 2A). Although we only used the AFM spherical probe images for training the deep learning model, we can see that the single-cell probe could be correctly recognized, which may be due to the similarity in shape between the microsphere tip and the cell tip (both round). On the basis of deep learning image recognition, the single-cell probe ( Figure 2C) was then precisely moved to the co-cultured MGC-803 cells ( Figure 2D) and HGC-27 cells ( Figure 2E) to perform an SCFS assay to measure the cellular adhesion forces. The real-time process of the SCFS assay by deep learning image recognition-assisted AFM is shown in Supplementary Movie S2. We measured 50 MGC-803 cells and 50 HGC-27 cells, and the statistical results (IV in Figure 2D,E) revealed that the adhesion force of HGC-27 cells was also greater than that of MGC-803 cells (statistical results for each cell line are shown in Supplementary Figure S5). Notably, we stained the co-cultured cells with fluorescein molecules to verify the detection results of the deep learning image recognition model. In the future, we can directly apply the trained deep learning image recognition model to identify co-cultured cell types in optical bright-field images without fluorescent labelling of the cells in advance.

**Figure 1:**
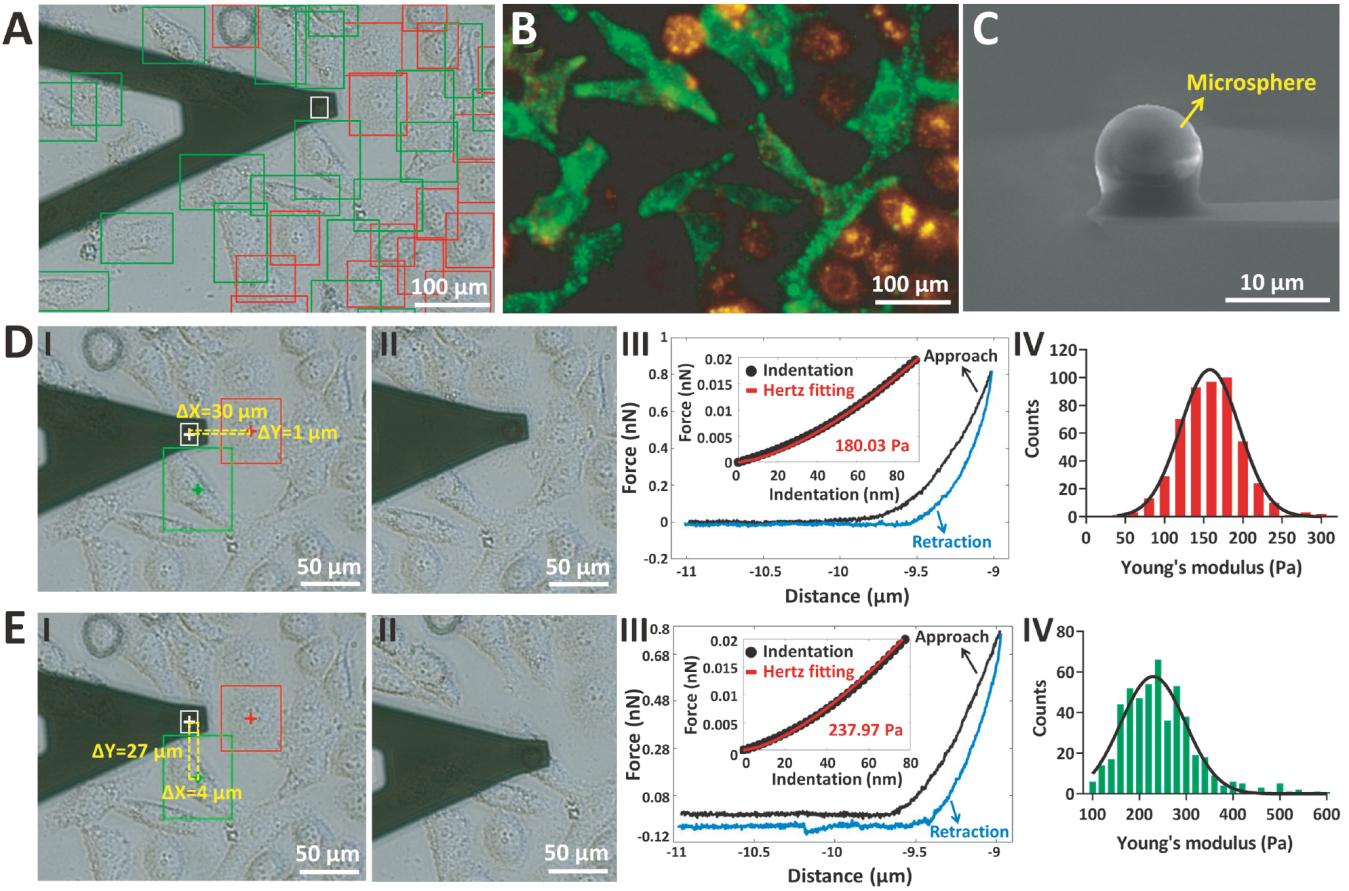
Figure 1 Indentation assay of co-cultured MGC-803 cells and HGC-27 cells by deep learning image recognition-assisted AFM using a microsphere-modified AFM probe (A,B) Optical bright-field image with deep learning detection results (the recognized MGC-803 cells are denoted by red boxes, the recognized HGC-27 cells are denoted by green boxes, and the AFM probe’s spherical tip is denoted by the white box) (A) and the corresponding fluorescent image (MGC-803 cells exhibit red fluorescence, and HGC-27 cells exhibit green fluorescence) (B). (C) Scanning electron microscopy (SEM) image of the prepared AFM spherical probe. (D,E) The AFM spherical probe was precisely moved onto a recognized MGC-803 cell (D) and a recognized HGC-27 cell (E) to perform the indentation assay. (I) The positional relationship (denoted by the dashed yellow box) between the AFM probe and the recognized cell. The centers of the target cells and the AFM spherical probe are marked by crosses inside the boxes. (II) Optical bright-field image showing that the AFM probe has been moved to the central area of the target cell. (III) A typical force curve obtained on the target cell with the Hertz model fitting result to extract the Young’s modulus of the cell (inset). (IV) Statistical results of the Young’s modulus measurements (the black curves are the Gaussian distribution fitting results). For the indentation assay, 50 MGC-803 cells and 50 HGC-27 cells were measured.

**Figure 2:**
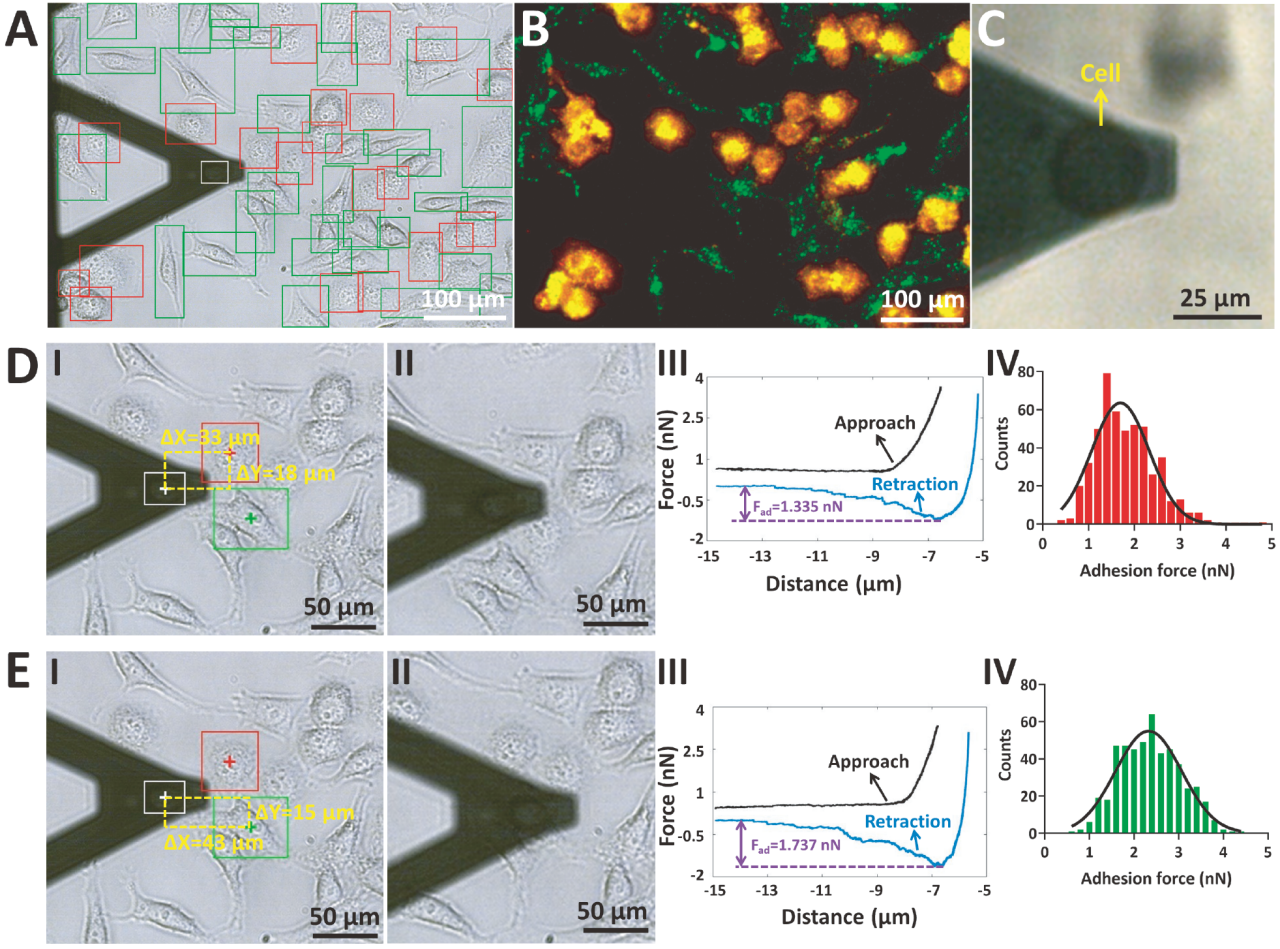
Figure 2 SCFS assay of co-cultured MGC-803 cells and HGC-27 cells by deep learning image recognition-assisted AFM using a single-cell probe A living HMrSV5 cell was attached to the AFM tipless cantilever. (A,B) Optical bright-field image with deep learning detection results (A) and the corresponding fluorescent image (B). (C) Optical bright-field image of a prepared AFM single-cell probe (the cell on the tipless cantilever is denoted by the yellow arrow). (D,E) The AFM single-cell probe was precisely moved to a recognized MGC-803 cell (D) and a recognized HGC-27 cell (E) to perform the SCFS assay. (I) Automatically determined positional relationship between the AFM probe and the recognized cell. (II) The AFM single-cell probe was moved to the target cell. (III) A typical force curve obtained on the target cell (the cellular adhesion force is denoted by the purple double arrow). (IV) Statistical results of the adhesion force measurements.

This study illustrates that deep learning image recognition-assisted AFM will facilitate the application of AFM-based force spectroscopy in the study of mechanobiology and cell-cell interactions. AFM has become an important research tool in the field of mechanobiology, but its practical application has long been limited by its low efficiency. The prerequisite of an AFM-based single-cell mechanical assay is positioning the tip of the AFM probe on the target cell, which currently relies mainly on manual experience. With deep learning image recognition-assisted AFM, the tip of the AFM probe can be precisely and rapidly moved to the target cells, which is independent of the experience of the operator. The automated single-cell mechanical measurement process by deep learning image recognition-assisted AFM was shown for both AFM spherical probes (used in the indentation assay to measure the cellular Young’s modulus) ( Figure 1) and single-cell probes (used in the SCFS assay to measure the cellular adhesion force) ( Figure 2), providing a feasible way to improve the throughput of AFM experiments for advancing the applications of AFM in mechanobiology. Notably, the central region of the cell was identified by the YOLOX model by annotating the entire cell in the optical bright-field image to generate the training data. In the future, by annotating only the local areas ( *e*. *g*., nuclei and peripheral zones) of cells to create training data, it will be possible to recognize the subcellular areas from optical bright-field images. Moreover, the utilization of a deep learning image segmentation model will further improve the precision of recognizing subcellular areas. In particular, physiological and pathological processes are often accompanied by interactions between different types of cells. For example, tumor formation and development involve interactions between cancer cells and microenvironmental cells ( *e.g.*, fibroblasts, monocytes, neutrophils, and platelets) [9]. Here, the experimental results show that the deep learning image recognition model can accurately identify the types of cells grown under high-density co-culture conditions from optical bright-field images, which allows AFM-based force spectroscopy to probe the mechanical properties of co-cultured cells in their native states (without fluorescent labelling), thereby facilitating the identification of new mechanical cues involved in cell-cell interactions to advance our understanding of life activities and human diseases [10].

Taken together, this work demonstrated the ability of deep learning image recognition-assisted AFM to measure the mechanical properties of individual living cells under high-density coculture conditions in an experience-free manner without the need for fluorescent staining, which will benefit the applications of AFM-based force spectroscopy in mechanobiology and facilitate the study of the mechanics of cell-cell interactions.

## Supporting information

23423Supplementary_materials

23423Supplementary_movie_2

23423Supplementary_movie_1

## Supplementary Data

Supplementary Data is available at *Acta Biochimica et Biophysica Sinica* online.
