# Supplementary material for: Label-free and rapid mechanics of single cells under high-density co-culture conditions by deep learning image recognition-assisted atomic force microscopy: Cell mechanics by deep learning image recognition-assisted AFM
Source: Acta Biochim Biophys Sin (Shanghai). 2024 Sep 18;57(2):317–20. doi: 10.3724/abbs.2024158 (PMC11868918; doi:10.3724/abbs.2024158)
Supplement: 23423Supplementary_materials [file 23423Supplementary_materials.pdf]

Supplementary materials

1. Video descriptions

Supplementary Movie S1. Measurement of the Young's modulus of single co-cultured cells (MGC-803 cells and HGC-27 cells) by deep learning image recognition-assisted AFM using the AFM probe carrying a microsphere

Supplementary Movie S2. Measurement of the adhesion force of single co-cultured cells (MGC-803 cells and HGC-27 cells) by deep learning image recognition-assisted AFM using the AFM probe carrying a living HMrSV5 cell

2. Materials and Methods

2.1 Cell sample preparation

The process of preparing co-cultured cell samples is as follows. Firstly, the DiI dye solution was added into the Petri dish containing MGC-803 cell monolayer and incubated for 30 min at 37 ℃ (5%CO_2 and 95% air). After the incubation, the DiI dye solution was removed from the dish and the dish was washed with phosphate-buffered saline (PBS) three times. The MGC-803 cells in the dish were then digested and suspended. The procedure for staining HGC-27 cells was the same as that for staining MGC-803 cells, except that the DiO dye solution was used for HGC-27 cells. Secondly, the stained MGC-803 cells and the stained HGC-27 cells were mixed at a ratio of 1:1 and added into a fresh Petri dish and incubated in the RPMI-1640 medium containing 10% fetal bovine serum (FBS) and 1% penicillin-streptomycin at 37 ℃ (5%CO_2 and 95% air) for 12 h. After the incubation, the cell culture medium was changed into the Leibovitz's L-15 medium for the subsequent experiments.

2.2 Deep learning image recognition model

The YOLOX deep learning neural network (downloaded from the GitHub website) and the PyTorch (v1.7.1) machine learning framework was used. For comparison, the same dataset was also used to train YOLOv5, YOLOv7 and YOLOv8. The annotation software LabelImg (downloaded from the GitHub website) was used to prepare the training data sets. With the use of LabelImg, MGC-803 cells and HGC-27 cells were annotated in the optical bright-field images (drawing the smallest box that can enclose the entire cell in the optical bright-field image) according to the corresponding fluorescent images. The AFM spherical probes were directly annotated in the optical bright-field images without the need of fluorescent images. Three types of data sets were prepared, including co-cultured MGC-803 cells (10118 cells were annotated), co-cultured HGC-27 cells (10180 cells were annotated), and AFM spherical probes (457 microspherical probe images were annotated). Each type of data set was divided into three categories, including training data set (accounting for 80% of the data set), validation data set (accounting for 10% of the data set), and test data set (accounting for 10% of the data set). The deep learning neural network was firstly trained with the public COCO data set (https://cocodataset.org/) for 90 epochs to obtain the pretrained weights. After that, the deep learning neural network was trained with the prepared data sets for 300 epochs (freezing training was used in the first 50 epochs, and unfreezing training was used in the next 250 epochs) using the pretrained weights. From the training results (**Supplementary Table S1**), we can see that YOLOX had the highest mean average precision (mAP) for the detection of the collected dataset, and thus the YOLOX model was used for the study.

Supplementary Table S1. Comparison of the training results of different YOLO series networks

Networks	Parameters	GFLOPs	FPS	mAP
YOLOX	54.150 M	155.318 G	22.77	89.25%
YOLOv5	46.149 M	107.964 G	27.02	88.14%
YOLOv7	37.205 M	104.795 G	28.29	85.82%
YOLOv8	43.632 M	165.112 G	22.34	88.75%

2.3 AFM experiments

The AFM spherical probes were prepared according to the previous protocol [1] for the indentation assay of co-cultured cells by deep learning image recognition-assisted AFM. The tipless cantilever (MLCT-010-C, Bruker, Santa Barbara, USA) with a spring constant of 0.01 N/m was used for preparing spherical probes. Microspheres (Baseline Company, Tianjin, China) with a diameter of 10 μm were used. Briefly, the microsphere solution was firstly dropped onto a coverslip until the microspheres on the coverslip were dry. The two parts of the epoxy glue (3M, Maplewood, MN, USA) were mixed and then coated evenly onto a bare area of the coverslip. Under the guidance of the optical microscopy, the AFM tipless cantilever was controlled to touch the glue, and the glue-coated cantilever was then controlled to adsorb a microsphere. The prepared AFM spherical probes were imaged by a scanning electron microscopy (SEM) (Thermo Fisher Scientific Company, Waltham, USA).

The AFM single-cell probes were prepared for the SCFS assay of co-cultured cells by deep learning image recognition-assisted AFM. The tipless cantilever (MLCT-010-E; Bruker, Santa Barbara, USA) with a spring constant of 0.1 N/m was used for preparing single-cell probes. Single living HMrSV5 cells were attached to the AFM tipless cantilever according to the previous protocol [2]. The reagents used in single-cell probe preparation were purchased from Solarbio Science & Technology (Beijing, China). Briefly, the tipless cantilever was modified with ConA molecules via the biotin-streptavidin system, and then the ConA-functionalized cantilever was used to adsorb a living HMrSV5 cell for subsequent SCFS assay.

For the AFM experiments, after moving the AFM probe to the vicinity of cells, an optical bright-field image was firstly obtained. The trained YOLOX model was then used to detect the optical bright-field image to identify the MGC-803 cells, HGC-27 cells and the AFM probe (spherical probe or single-cell probe) in the optical bright-field image, and automatically determine the positional relationships between the AFM probe and the target cells. After inputting the positional relationships to the instrumental software of the AFM (JPK NanoWizard Control Software, Bruker, Santa Barbara, CA, USA), the AFM probe was accurately moved to the target cells to perform force measurements. For SCFS assay, HMrSV5 cell suspension solution was added to the dish containing MGC-803 cells and HGC-27 cells for the preparation of single-cell probes. The parameters of obtaining force curves on MGC-803 cells were the same as those obtained on HGC-27 cells to make the results comparable. For indentation assay, 50 MGC-803 cells and 50 HGC-27 cells were measured. For SCFS assay, 50 MGC-803 cells and 50 HGC-27 cells were measured. Since the AFM spherical probe was used, the Young's modulus values of cells were extracted from the force curves by applying Hertz model [3]:

$$F = \frac{4E\delta^{1.5}\sqrt{R}}{3(1-\upsilon^2)} \quad (1)$$

where υ is the Poisson ratio of cell being measured by AFM (cells are often considered as incompressible materials and thus υ=0.5 for cells), δ is the indentation depth, E is the Young's modulus of the cell being measured, R is the radius of the microsphere on the AFM probe, and F is the loading force. According to the contact point in the approach curve, the approach curve was converted into the indentation curve. The indentation curve was then fitted with the Hertz model by the program written by the software Matlab (MathWorks, Natick, USA).

References

1. Yang X, Yang Y, Zhang Z, Li M. Deep learning image recognition-assisted atomic force microscopy for single-cell efficient mechanics in co-culture environments. Langmuir 2024, 40: 837-852.

2. Friedrichs J, Helenius J, Muller DJ. Quantifying cellular adhesion to extracellular matrix components by single-cell force spectroscopy. Nat. Protoc 2010, 5: 1353-1361.

3. Li M, Xi N, Wang Y, Liu L. Atomic force microscopy in probing tumor physics for nanomedicine. IEEE Trans. Nanotechnol. 2019, 18: 83-113.

3. Supplementary Figures

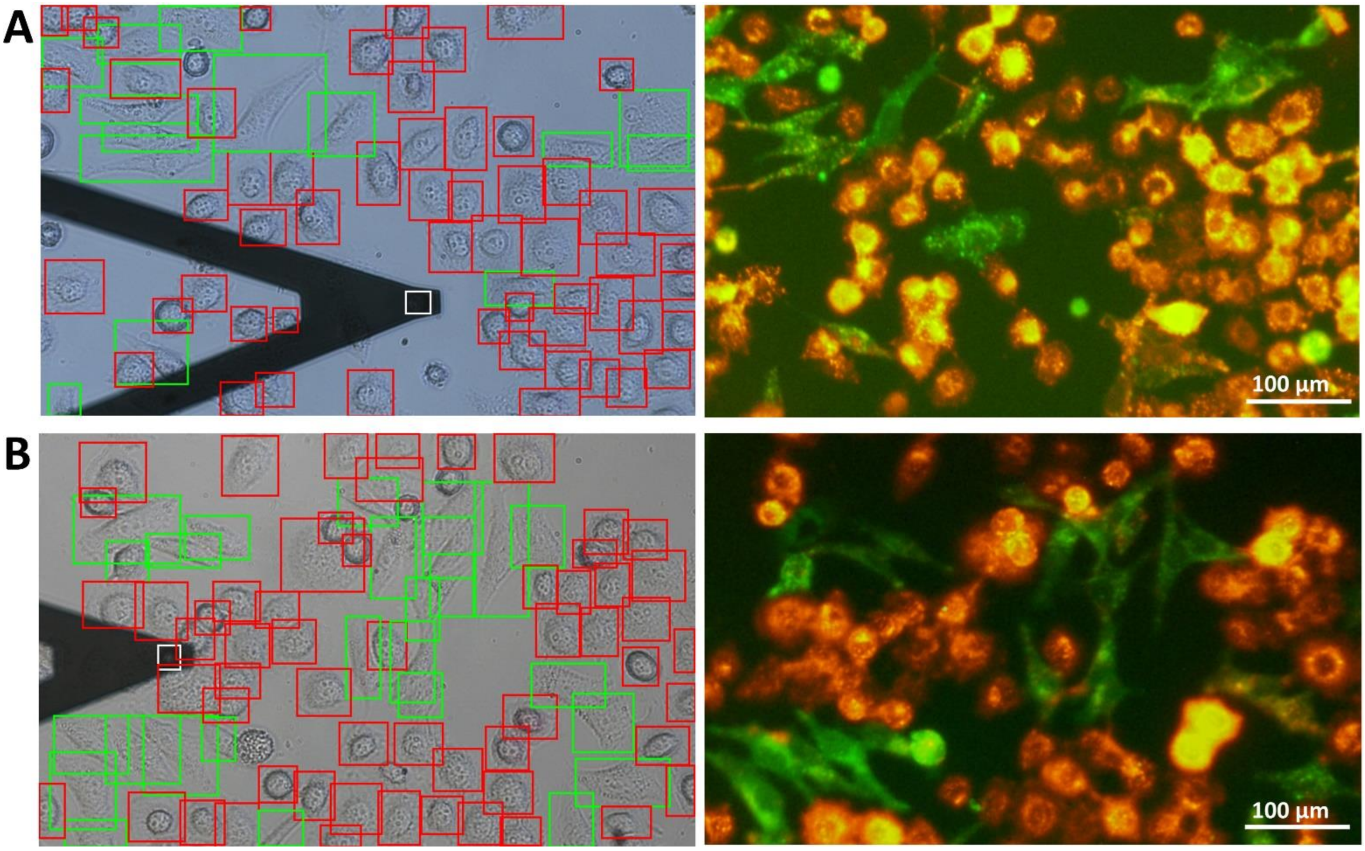

Supplementary Figure S1. Preparation of the training datasets (A) An optical bright-field image with box labels (left) and the corresponding fluorescent image (right). (B) Another optical bright-field image with labels (left) and the corresponding fluorescent image (right). The red box labels indicate MGC-803 cells, the green box labels indicate HGC-27 cells, and the white box labels indicate the spherical tip of the AFM probe. The labels were generated according to the results of the fluorescent images (except for AFM probes). The optical bright-field images with the labels were then used for training of the deep learning model.

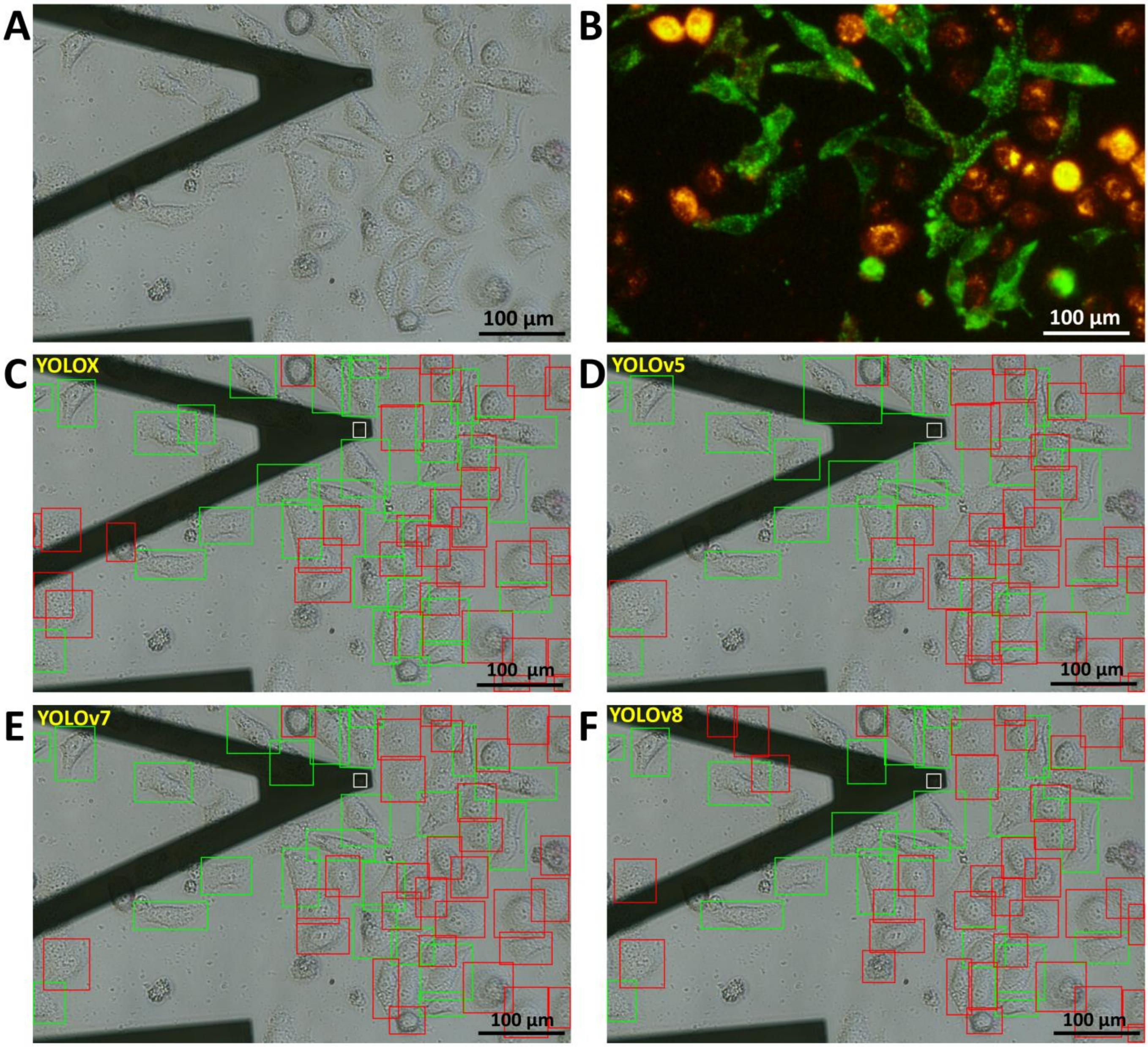

G

Networks	Total number of cells detected	Number of cells recognized correctly	Number of cells recognized incorrectly
YOLOX	57	57	0
YOLOv5	53	48	5
YOLOv7	51	49	2
YOLOv8	54	52	2

Supplementary Figure S2. Comparison of the four YOLO networks for recognizing co-cultured cells in the optical bright-field image (A,B) Optical bright-field image (A) and the corresponding fluorescent image (B). (C–F) Cell recognition results of the four YOLO networks. (C) YOLOX. (D) YOLOv5. (E) YOLOv7. (F) YOLOv8. (G) Statistical results.

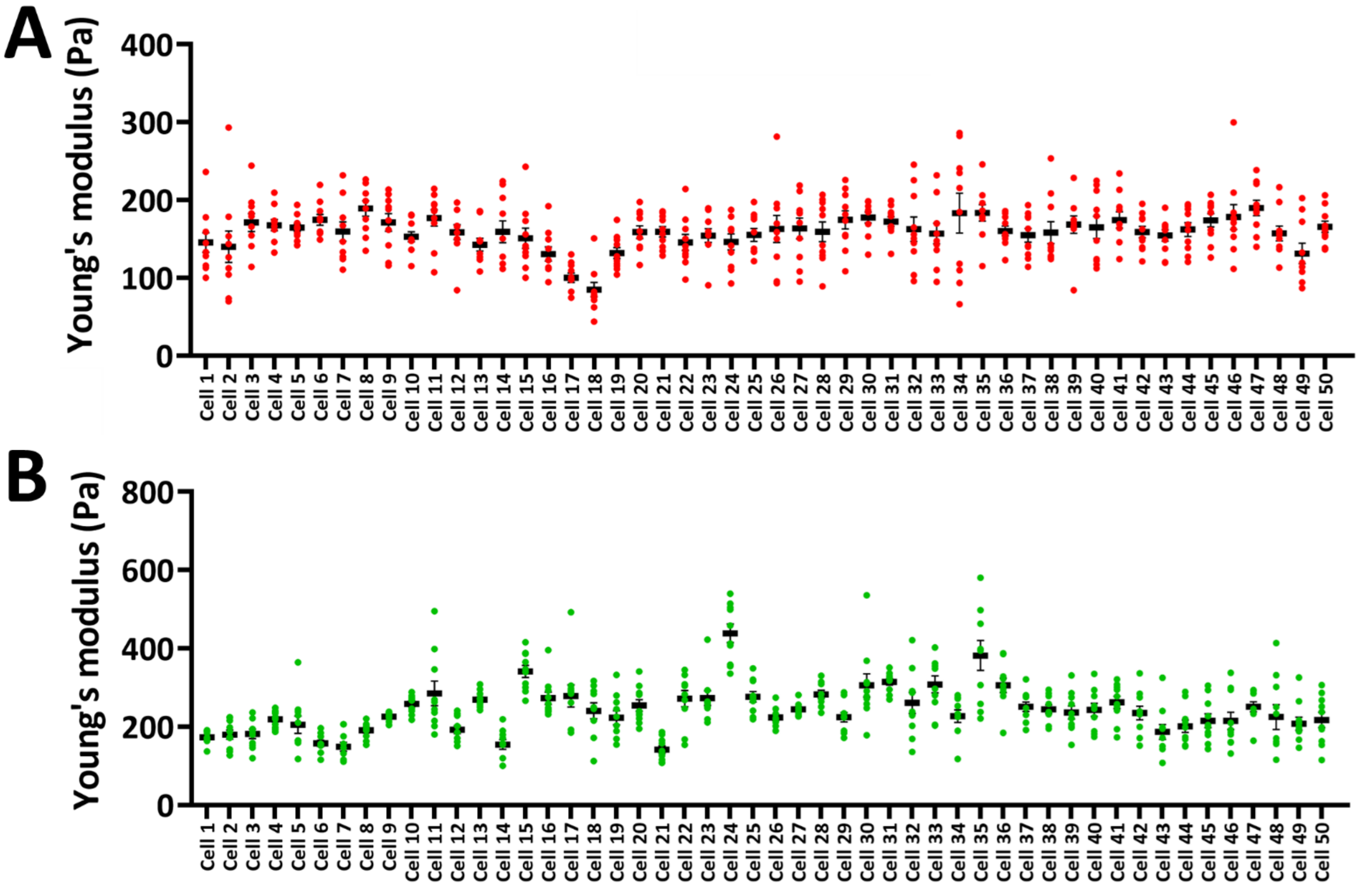

Supplementary Figure S3. Scatter plots of the Young's modulus values of 50 MGC-803 cells (A) and 50 HGC-27 cells (B) measured by deep learning image recognition-assisted AFM The black bars indicate the mean values and the error bars show the SEM.

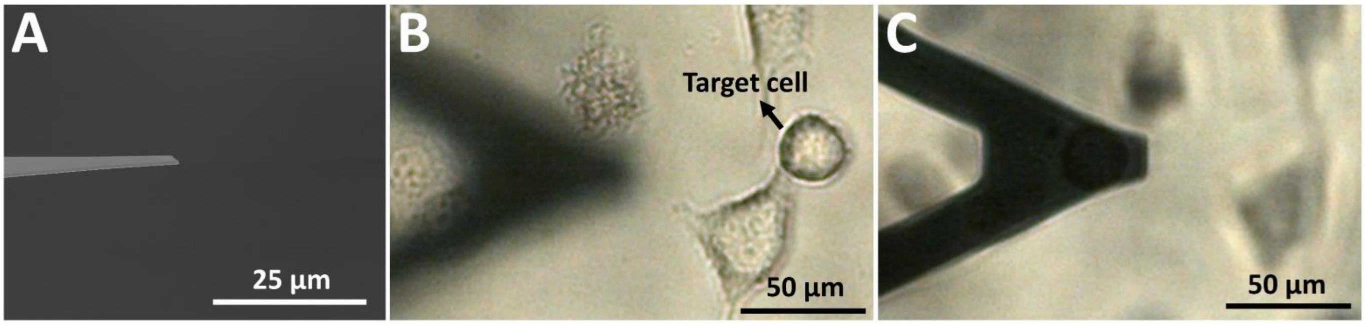

Supplementary Figure S4. Preparing the single-cell probe for SCFS assay (A) SEM image of an AFM tipless cantilever used for the preparation of single-cell probe. (B,C) Optical bright-field images showing the preparation of a single-cell probe. (B) The AFM tipless cantilever coated with ConA molecules *via* the biotin-streptavidin system is controlled to adsorb a living HMrSV5 cell (denoted by the black arrow). (C) The target HMrSV5 cell has been attached to the AFM tipless cantilever.

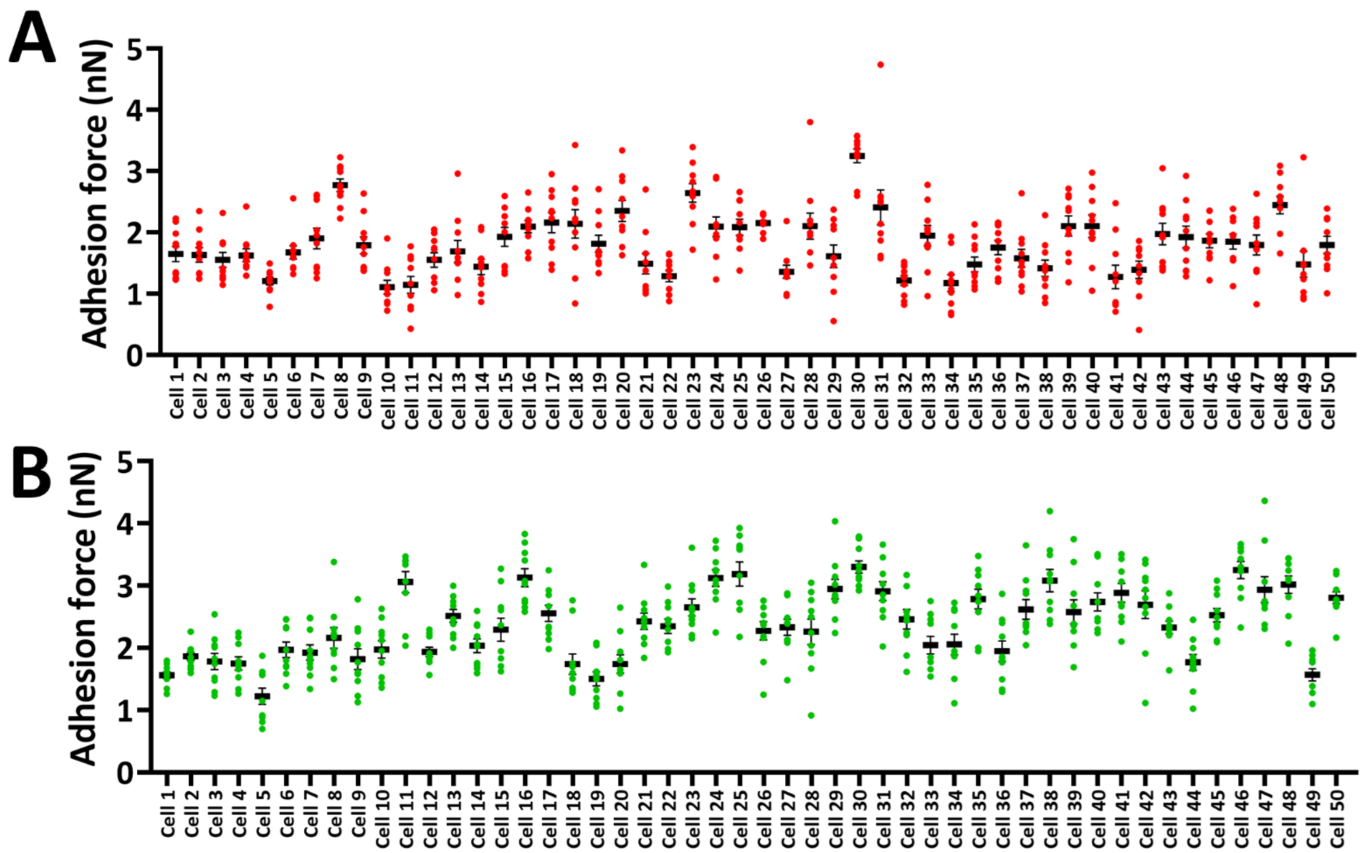

Supplementary Figure S5. **Scatter plots of the adhesion forces of 50 MGC-803 cells (A) and 50 HGC-27 cells (B) measured by deep learning image recognition-assisted AFM** The black bars indicate the mean values and the error bars show the SEM.
